# Advancement in liver laparoscopic resection – development of a new surgical device

**DOI:** 10.1590/1414-431X20176062

**Published:** 2018-03-01

**Authors:** P. Vavra, L. Karnik, M. Skrobankova, J. Jurcikova, P. Ihnat, P. Zonca, M. Peteja, A. El-Gendi, S. Czudek

**Affiliations:** 1 Department of Surgical Studies, Faculty of Medicine, University of Ostrava, Ostrava, Czech Republic; 2 Department of Robotic, Faculty of Mechanical Engineering, VSB–Technical University of Ostrava, Ostrava, Czech Republic; 3 Department of Surgery, University Hospital Ostrava, Ostrava, Czech Republic; 4 Department of Vice-President for Science and Research, University Hospital Ostrava, Ostrava, Czech Republic; 5 Department of Cybernetics and Biomedical Engineering, Faculty of Electrical Engineering and Computer Science, VSB–Technical University of Ostrava, Ostrava, Czech Republic; 6 Department of Surgery, Faculty of Medicine, Alexandria University, Alexandria, Egypt; 7 Department of Internal Medicine, University Hospital of Ostrava, Ostrava, Czech Republic

**Keywords:** Laparoscopic surgery, Liver, Radiofrequency energy, Posterior liver segments

## Abstract

Liver resection is the standard treatment for any liver lesion. Laparoscopic liver resection is associated with lower intra-operative blood loss and fewer complications than open resection. Access to the posterior part of the right liver lobe is very uncomfortable and difficult for surgeons due the anatomic position, especially when employing laparoscopic surgery. Based on these experiences, a new laparoscopic device was developed that is capable of bending its long axis and allowing the application of radiofrequency energy in areas that were not technically accessible. The device is equipped with four telescopic needle electrodes that cause tissue coagulation after the delivery of radiofrequency energy. *Ex vivo* testing was performed in 2012 and 2014 at the University Hospital, Ostrava, on a porcine liver tissue. The main goal of this testing was to verify if the newly proposed electrode layout was suitable for sufficient tissue coagulation and creating a safety zone around lesions. During the *ex vivo* testing, the material of needle electrodes was improved to achieve the lowest possibility of adhesion. The power supply was adjusted from 20 to 120 W and the ablation time, which varied from 10 to 110 s, was monitored. Subsequently, optimal power delivery and time for coagulation was determined. This experimental study demonstrated the feasibility and safety of the newly developed device. Based on the *ex vivo* testing, LARA-K1 can create a safety zone of coagulation. For further assessment of the new device, an *in vivo* study should be performed.

## Introduction

Hepatic resection is currently the standard treatment for liver lesions. The number of liver resections is increasing due to increasing numbers of primary and secondary tumors of liver parenchyma. Secondary tumors and metastases in liver are mainly caused by gastrointestinal tumors, most often by colorectal cancer. The incidence of colorectal cancer is still increasing globally and thus the number of liver metastases is increasing as well. Liver resection is the first-line treatment option for patients with primary and secondary liver tumors (1). Laparoscopic liver resection is common and an easier liver surgery technique performed in the recent times.

Radiofrequency energy is currently employed in the treatment of numerous medical indications. Radiofrequency is a high frequency alternating electrical current that creates the desired clinical effect by passing through the tissue. It is capable of heating the tissue around the active electrodes (2,3). Radiofrequency energy has been used in medicine since the 19th century (4). Radiofrequency ablation has been incorporated into liver surgery for many years and has become one of the standard methods in the treatment of primary and secondary liver malignancies (5). There are many studies that document the use of radiofrequency in different ways to improve treatment protocols (6,7). Radiofrequency-assisted liver resection represents a safe and effective way of hepatic parenchyma transection (8). The radiofrequency assisted resection technique is reported to be associated with minimal blood loss, low blood transfusion requirement, and reasonable postoperative morbidity and mortality (9).

Many studies have compared laparoscopic and open surgery and reported that laparoscopic liver resection is associated with lower intra-operative blood loss and fewer complications and also laparoscopic hepatectomy has similar short-term outcomes compared to open hepatectomy (10). In cases of superficial and sub-superficial liver lesions of anterior segments, laparoscopic resection can be indicated as well as open surgery. There is no difference in survival rates after resection of colorectal liver metastases by laparoscopic or open surgery (11). Laparoscopic resections are better tolerated, especially by patients with liver cirrhosis (12).

According to anatomical division, segments VII and VIII were traditionally considered unsuitable for the laparoscopic approach; however, Cho et al. (13,14) reported a series of 36 patients with lesions in the postero-superior segments, who underwent laparoscopic liver resections. Modern surgery is trying to be as minimally invasive as possible. Thus, efforts are being made to adapt the laparoscopic approach to an otherwise non-resectable tumor of the posterior segments. The segments of the right posterior lobe (VII and VIII) are difficult to access for laparoscopic liver resection. The new proposed laparoscopic device can simplify the access to those segments and aid non-anatomical resections.

Based on the above information, a new device was developed, which facilitated access to the posterior segments in the laparoscopic approach. Authors worked on two projects using radiofrequency energy for development of the new instrument, registered at the "Patent Protection of Industrial Property". Two successful *in vivo* studies on porcine models were also performed in cooperation with University of Veterinary and Pharmaceutical University Brno (15).

The main purpose of this *ex vivo* study was to determine if the prototype of the newly developed adjustable device (LARA-K1) is functional and safe.

## Material and Methods

**Figure 1.**
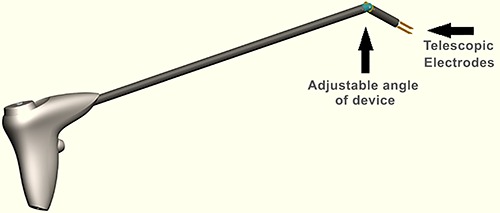
LARA-K1. Functional prototype with angled end in the long axis.

The laparoscopic device was designed to fit the commonly used laparoscopic instruments. At the end of the long axis of the device are four telescopic electrode needles. These needles, manufactured from special structural steel, can cause coagulation of the liver tissue after the delivery of radiofrequency energy. The joint, which can bend into a different angle, is located along the long axis of the device (Figure 1).

With the telescopic needles and adjustable angle of use, this device can readily access the posterior segments. Two rotating buttons allow very simple manipulation of the adjustable possibilities. With the first button, we can eject the four electrodes (needles) from 0 to 0.03 m and the second button flexes the axis in angles from 0^o^ to 60^o^. The connection with the current generator of radiofrequency energy is considered a potential advantage. There are no requirements for additional technical instruments.

**Figure 2.**
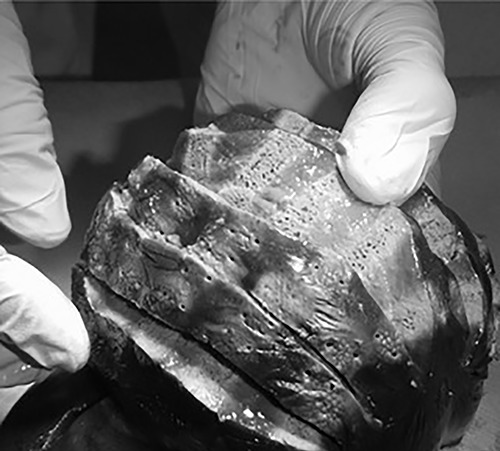
*Ex vivo* study. Application of the new laparoscopic device caused coagulation necrosis in the liver tissue.

The initial phase of development included putting together a set of medical requirements for the laparoscopic instrument and technical possibilities. During 2009–2012, a new device was developed, which, after its introduction into the abdominal cavity through a laparoscopic trocar, could bend its long axis and eject the telescopic needles to the required position. This technical arrangement allows the application of radiofrequency energy in areas that are currently inaccessible. The technical construction of the prototype was performed by experts from the Department of Robotics of the Technical University of Ostrava (Figure 2).

**Figure 3.**
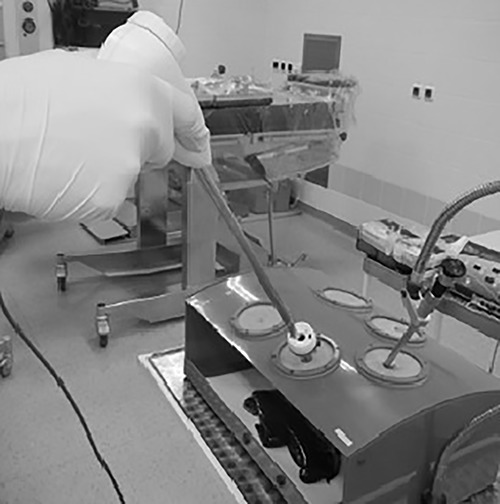
*Ex vivo* study. Use of laparoscopic simulator for the evaluation of the manipulation of the new device.

*Ex vivo* testing was performed in 2012–2014 in the experimental surgery theater at the University of Ostrava on porcine liver tissue because of the similarities in structure to the human liver. The main goal of the testing was to evaluate if the newly proposed electrode layout was suitable for sufficient tissue coagulation, creating a safety zone around the lesion. Another goal of the study was to discover any potential faults. The laparoscopic simulator was used to determine the accuracy of manipulation (Figure 3).

During the *ex vivo* testing, settings of power delivery, depth of the puncture of electrodes, time required to create a safety zone of coagulation necrosis, and the adhesiveness of electrode needles were monitored.

The instrument was connected to a commonly used radiofrequency generator. Thermal camera FLUKE Ti25 (Fluke Corporation, USA) was used to control the thermal distribution of this device.

## Results

During the *ex vivo* testing, many small objectives were accomplished. The material of the electrode needles was improved to structural steel with a special coating, which can decrease the adhesiveness of electrodes to the zero point. The optimal power delivery from the commonly used radiofrequency generator was established. The time required to coagulate the liver tissue depended on the delivered power. Many trials were performed to set the time of coagulation according to the power supply. Ablation time was from 10 to 110 s and power supply was from 20 to 120 W. The optimal delivery power and time, which are required to create a safety zone of coagulation necrosis in liver parenchyma, was determined.

The optimal delivery power from the standard radiofrequency generator was set to 90 W with a time of 14 s on average to sufficiently coagulate the liver tissue. The optional material for electrodes is structural steel with a special coating, which decreases the adhesiveness as much as possible.

## Discussion

This study was based on the application of knowledge about radiofrequency energy for practical use in laparoscopic liver surgery. A newly adjustable laparoscopic device was created, and an *ex vivo* study was performed for proof of efficiency. This laparoscopic technique allows the performance of a larger number of smaller liver resections with minimal blood loss and less effect on the "healthy" liver parenchyma (16,17).

Studies (18–20) confirmed significantly less blood loss in laparoscopic liver surgery compared to laparotomy. Another study with a large number of laparoscopically operated patients showed the need for blood transfusion in 0.7% compared to 8% in open surgery (21). The most frequent indications for laparoscopic liver resection are lesions in segments II–VI, especially for wedge resection, segmentectomy or lateral left-side hemihepatectomy. Liver segments I, VII, and VIII are traditionally considered inaccessible for laparoscopic approach due to their anatomical position in relation to the other structures.

This experimental study demonstrated the feasibility and safety of the newly developed laparoscopic adjustable device. Testing the device on liver without blood flow and other properties of the *in vivo* liver are potential limitations. Therefore, it is necessary to test the device in an *in vivo* study on laboratory animals. By using the laparoscopic simulator, we confirmed that manipulation with the newly designed device LARA-K1 is satisfactory in the liver with blood flow.
